# Human MHC-II with Shared Epitope Motifs Are Optimal Epstein-Barr Virus Glycoprotein 42 Ligands—Relation to Rheumatoid Arthritis

**DOI:** 10.3390/ijms19010317

**Published:** 2018-01-21

**Authors:** Nicole Trier, Jose Izarzugaza, Anna Chailyan, Paolo Marcatili, Gunnar Houen

**Affiliations:** 1 Department of Autoimmunology and Biomarkers, Statens Serum Institute, Artillerivej 5, 2300 Copenhagen, Denmark; 2 Department of Bioinformatics, Technical University of Denmark, Anker Engelundsvej 1, 2800 Kongens Lyngby, Denmark; txema@bioinformatics.dtu.dk (J.I.); pamar@bioinformatics.dtu.dk (P.M.); 3 Carlsberg Research Laboratory, J. C. Jacobsens Gade, 1799 Copenhagen, Denmark; anna.chailyan@gmail.com

**Keywords:** Epstein-Barr virus, glycoprotein 42, rheumatoid arthritis, shared epitope

## Abstract

Rheumatoid arthritis (RA) is a chronic systemic autoimmune disorder of unknown etiology, which is characterized by inflammation in the synovium and joint damage. Although the pathogenesis of RA remains to be determined, a combination of environmental (e.g., viral infections) and genetic factors influence disease onset. Especially genetic factors play a vital role in the onset of disease, as the heritability of RA is 50–60%, with the human leukocyte antigen (HLA) alleles accounting for at least 30% of the overall genetic risk. Some HLA-DR alleles encode a conserved sequence of amino acids, referred to as the shared epitope (SE) structure. By analyzing the structure of a HLA-DR molecule in complex with Epstein-Barr virus (EBV), the SE motif is suggested to play a vital role in the interaction of MHC II with the viral glycoprotein (gp) 42, an essential entry factor for EBV. EBV has been repeatedly linked to RA by several lines of evidence and, based on several findings, we suggest that EBV is able to induce the onset of RA in predisposed SE-positive individuals, by promoting entry of B-cells through direct contact between SE and gp42 in the entry complex.

## 1. Introduction

### 1.1. Rheumatoid Arthritis

Rheumatoid arthritis (RA) is a chronic systemic autoimmune disease of unknown etiology. If left untreated, the disease manifests as sustained synovitis and erosions of articular cartilage and surrounding bone, which causes joint damage, reduced mobility and decreased quality of life, as well as cardiovascular and other extra-articular complications [1,2]. The typical clinical presentation of RA is a symmetrical peripheral joint arthritis and progressive erosions of the affected joints [1,2]. The disease course of RA is highly variable; the course and the severity of the arthritis may vary from quite mild to extremely destructive, resulting in severe disability. Thus, in a limited group of RA individuals, the arthritis is self-limiting, however, most patients suffer from chronic arthritis. Besides causing significant clinical problems, RA is also responsible for substantial economic and social costs, particularly from work-related disability [3].

RA affects approximately 1–2% of the world’s population with 5–50 new cases per 100,000 individuals annually [4,5]. The disorder is most typical in elderly people and women, with a female preponderance of 3:1 [6,7], and onset of the disease is most frequent between the ages of 40–50 [5], suggesting that hormonal factors could have a pathogenic role [7].

RA is diagnosed according to clinical manifestations supported by detection of the autoantibodies IgM/IgA rheumatoid factor (RF) and anti-citrullinated protein antibodies (ACPA) [8]. Being specific for the Fc region of IgGs, RFs are detected in approximately 50–90% of RA individuals, dependent on age [9,10,11]. Approximately 70–80% of RA individuals are ACPA positive, and as with RF, these antibodies are present early in the course of the disease and precede clinical onset [12,13,14,15]. Compared to RFs, ACPA are more RA-specific, as RFs also may be detected in individuals affected by infections, other autoimmune diseases, e.g., such as systemic lupus erythematosus (SLE), mixed connective tissue disease, Sjögren’s syndrome, and occasionally in healthy individuals [16,17].

Antibodies recognizing epitopes with the modified amino acid residue citrulline (Cit), are referred to as ACPAs. These antibodies are primarily directed to citrullinated proteins located in the joints [18,19]. Citrullination, catalyzed by the calcium-dependent peptidyl arginine deiminase (PAD) enzymes, is a post-translational modification of arginine generated as a result of deimination [20], which physiologically occurs during apoptosis, inflammation or keratinization [21]. Under pathological conditions, where cell death may overwhelm the phagocytic capacity of phagocytes, necrotic cells may release PAD into the extracellular space, where higher calcium concentrations allow citrullination of other proteins located outside the cell [21]. Therefore, when the apoptotic cells are not cleared efficiently, such as in an inflammatory environment, intracellular proteins and/or PAD are released into the extracellular space, where the former are taken up by antigen-presenting cells and the latter induces citrullination of synovial joint proteins. Consequently, antibodies to various citrullinated proteins are locally produced in affected joints, where proteins are citrullinated during the inflammatory process [22]. Interestingly, ACPAs have been proposed to be involved in the pathogenesis of RA, although no exact mechanism has been determined [12,23].

Through the identification and characterization of ACPAs, and by novel insights into RA-diagnosis and etiopathology, it has become clear that RA is of heterogeneous nature, consisting of clinical subsets of ACPA-positive and ACPA-negative RA. These subsets share many clinical features, but differ with respect to genetic background, predisposing environmental factors and clinical progression/remission [14,24,25,26]. Consequently, individuals with ACPA-positive RA typically have severe symptoms and disease course, whereas individuals with ACPA-negative RA often experience a mild disease course [24,27,28,29].

### 1.2. Rheumatoid Arthritis and Genetic Risk Factors

Based on twin studies, it has been proposed that the relative contribution of genetic variation to the liability of developing RA is around 60% [2,30]. The strongest evidence for the influence of genetic factors on RA onset relates to major histocompability complex (MHC) class II antigens, and, in particular to various human leukocyte antigen (HLA) alleles, e.g., HLA-DR. HLA-DR is a MHC cell-surface receptor, which interacts with T-cell receptors through presentation of internalized antigens, which ultimately results in stimulation of T-cells and antibody-producing B-cells. Widely recognized alleles that are major contributors to RA risk at the DRB1 locus are DRB1*04:01, *04:04, *04:05, *04:08, 04:09, *01:01, *01:02, *10:01 and *14:02 (Table 1) [31].

**Figure 1:**
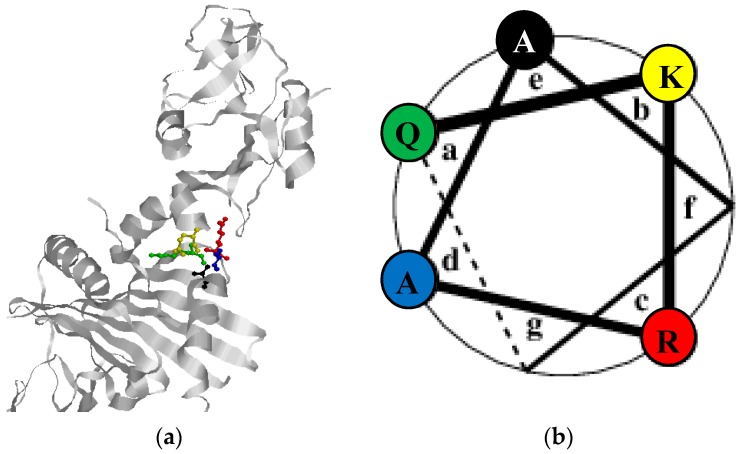
Structure analysis of the shared epitope motif in the HLA-DR1-gp42 complex. (**a**) The SE motif (amino acids 70–71) is located in an α-helix structure. The individual amino acids and their orientation is visualized by the following colors Q70 (green), K71 (yellow), R72 (red), A73 (blue), A74 black. A, K, Q, R represent the amino acids Ala, Lys, Gln and Arg. (**b**) Helical wheel of the SE motif. The left side of the wheel faces the peptide groove, the right side is on the “outside” of the helix. The heptad positions of the helix are labeled a–g, by convention.

All of these HLA-DRB1 alleles share a common amino acid motif, referred to as the shared epitope (SE) [34]. In fact, it has been estimated that up to 50% of RA patients are positive for this amino acid motif [35]. Stastny originally documented an association between HLA-DR4 and the risk of developing RA [34]. Discrepancy in the association of different HLA-DRB1 genes revealed the presence of a conserved hexameric amino acid sequence in the third hypervariable regions of all RA-associated HLA-DRB1 alleles, involving amino acid positions 70–74 and consisting of glutamine (arginine), lysine (arginine), arginine, alanine and alanine “R/QK/RRAA”, also referred to as the SE structure [34,35], although the most common sequence of amino acids in these positions is QKRAA. These residues constitute an α-helix (Figure 1), forming one side of the antigen-binding cleft, a site likely to affect antigen presentation. Especially position 70 of the SE has received attention, as glutamine or arginine in position 70 are critical for the risk of developing RA, whereas aspartic acid in that position appears to have a protective effect [36]. Although the SE structure is conserved in some alleles, further differentiations in the third hyper-variable region have been proposed. For example, HLA-DRB1 alleles can be discriminated in the amino acid region from 71 to 86 [37]. Other studies propose another classification focusing primarily on the positions 72–74 (RAA), which is modulated by the amino acid in position 71 (K confers the highest risk, R an intermediate risk, E and A a lower risk) and by the amino acid in position 70 (R or Q confers a higher risk than D) [33,38].

In addition to prior indications that aspartic acid in position 70 may reduce RA risk, it also appears to reduce disease severity. By analyzing the effect of the DERAA sequence (residues 70–74 encoded by several HLA-DRB1 alleles, including the RA-protective HLA-DRB1*04:02 allele) on disease outcomes in individuals with early arthritis, it has been found that in RA patients without early erosions, DERAA-coding DRB1 alleles are strongly protective against severe disease [39]. Similarly, alleles carrying Ile in position 67 appear to have a protective effect [40], whereas variants at position 11 and 13 in DRB1 have been proposed to predispose strongly to RA as well [41,42]. Furthermore, alleles such as HLA-DRB1*11:01, *11:04, *12:01 and *16:01 have been reported to be correlated with benign forms of RA [32].

Among the SE alleles, DRB*04:01 and *04:04 confer a stronger disposition to RA than DRB1*01:01 and *10:01 [40,42]. Similarly, DRB1*04:01 homozygosity and DRB1*04:01/*04:04 heterozygosity are associated with increased risk for RA [40]. The associations between HLA and RA have been analyzed mainly for the DR loci. However, the strong linkage disequilibrium between DR and DQ suggests that both DR and DQ may contribute to predisposition to RA. 

Besides causing a predisposition to RA, the SE motif has been proposed to promote joint destruction and extra-articular involvement and even early mortality [43,44]. Interestingly, in Europeans, the association between DRB1 and RA is stronger in ACPA-positive RA than in ACPA-negative RA [15,40,41]. Thus, in RA individuals with heterozygosity and homozygosity of HLA-DRB1 SE alleles, ACPA production has been found to be significantly increased [15,40,41]. Similarly, the risk of developing RA is reduced in SE-negative individuals, although it has been proposed that exposure to maternal antigens (e.g., HLA molecules) in utero could contribute to RA development in SE-negative women [35]. 

The mechanism underlying SE-positive RA remains unclear [45,46,47,48,49]. It has been hypothesized that SE-positive DRB1 alleles confer disease susceptibility through a mechanism that involves alteration of the peripheral T-cell repertoire or through the selective presentation of arthritogenic self or foreign peptides [45,46,47,48,49]. In addition, it has been described that the DRB1*04:01 protein interacts with citrullinated peptides with higher affinity than with non-citrullinated peptides, which may indicate that the SE alleles exert pathogenic effects through the presentation of citrullinated peptides, which are recognized as non-self by T-cells [50]. Similarly, it has been found that the citrullinated DERAA motif, which is found in DRB1 alleles, including DRB1*13 may have a protective function [51]. This protective effect is, among others, ascribed to the cross-reactivity of self-reactive T-cells to the citrullinated motif [51]. Finally, it has been proposed that the SE, analogous to certain domains of class I MHC-molecules [52,53], acts as a ligand that interacts with cell surface calreticulin and activates innate immune signaling [54]. However, the exact role of SE in the onset of RA remains to be determined. 

The second major polymorphism occurs in the *PTPN22* gene, which encodes the protein tyrosine phosphatase, non-receptor type 22, a tyrosine phosphatase of importance in T-cell signaling [55,56]. Interestingly, this gene is a genetic risk factor in other autoimmune diseases as well, e.g., the onset of type 1 diabetes, which correlates with an increased risk of developing type 1 diabetes in ACPA-positive RA individuals. 

In general, the currently known genetic risk factors associated with RA are thought to be specifically associated with either ACPA-positive or ACPA-negative disease. Thus, ACPA-positive RA has been found to be closely linked to the presence of HLA-DRB1 alleles containing SE motifs [57,58] and polymorphisms in the *PTPN22* gene [56,57,59]. Moreover, ACPA-positive status has been suggested to be associated with the recently identified, but modest genetic risk factor tumor necrosis factor receptor-associated factor 1 (TRAF1)-C5 [60]. Other genetic factors such as variations in the interferon-regulating factor (IRF)-5 and polymorphisms in a newly identified risk gene in the C-type lectin complex have been suggested to be associated with ACPA-negative RA disease [61,62].

Additional genetic risk factors have been proposed, including PAD4, signal transducer and activator of transcription (STAT4), cluster of differentiation 244 (CD244) and cytotoxic T lymphocyte-associated antigen 4 (CTLA4), located outside the MHC [63].

### 1.3. Rheumatoid Arthritis and Environmental Risk Factors

Various environmental factors have been linked to the onset of RA, e.g., infectious agents and smoking [64,65,66]. Among several environmental factors, which are implicated in the onset of RA, infectious agents have been suggested to be the most likely culprits [65]. A variety of viral candidates has been proposed, e.g., Epstein-Barr virus (EBV), Parvovirus B19 and Rubella virus. Moreover, some bacterial candidates have been linked to the onset of RA as well, e.g., *Proteus mirabillis* [65] and *Porphyromonas gingivalis* [67]. The latter are both gram-negative anaerobic bacteria, but *Proteus mirabillis* is primarily associated with urinary tract infection, whereas *P. gingivalis* primarily is associated with periodontal disease. Interestingly, *P. gingivalis* is the only bacterium known so far to contain a PAD enzyme, which is involved in citrullination of both bacterial and human proteins in periodontal tissue [68,69]. Moreover, RA is prevalent in individuals with chronic periodontitis [70]. Based on these findings it has been suggested that *P. gingivalis* can potentially contribute to the generation of de novo epitopes that may trigger the formation of ACPA. Several reviews nicely illustrate the connection between RA, ACPA and bacterial PAD [71,72]. Nevertheless, contradictory data have been published regarding the correlation between the levels of antibodies against *P. gingivalis* and ACPA in RA individuals [69,73,74]. ACPA might be produced outside the joint in mucosal sites such as the lung and gingiva. Consequently, ACPA might cross-react through molecular mimicry with citrullinated epitopes in the joint initiating an inflammatory response in genetically susceptible individuals. Cigarette smoking constitutes the main environmental risk for development of RA. It is well established that cigarette smoking significantly increases the risk of RA [75,76,77]. Although it remains to be determined exactly how cigarette smoking induces the onset of RA and the pathogenic effect of smoking, several mechanisms have been proposed to understand the role of cigarette smoking in RA [75,76,77]. Smoking is known to modulate the immune system through many mechanisms, including the induction of the inflammatory response, immune suppression, alteration of cytokine balances and induction of apoptosis. In addition, recent studies ascribe an inhibitory effect of smoking on RA treatment, as the response and drug survival in RA patients treated with anti-tumor necrosis factor therapy is reduced in heavy smokers [78]. No sole mechanism, however, has been linked to RA, which therefore complicates full comprehension of the smoking effect [75]. A profound gene-environment interaction between smoking and HLA-DR SE genes as risk factors is evident. In individuals who are HLA-DR SE-negative, smoking is a relatively modest risk factor, however, in individuals who carry one or two sets of the SE genes, smoking dramatically increases the risk of developing RA [71,79]. A similar picture applies to the risk of developing ACPA-positive RA, although the risk primarily applies to individuals having two sets of the SE alleles [80]. A report from the Swedish population-based case-control study Epidemiologic Investigation of Rheumatoid Arthritis (EIRA), in which RA cases are recruited within one year of disease onset, found that smokers, who do not carry the SE, have a 1.5-fold elevated risk of developing ACPA-positive RA over non-smokers, who also do not carry the SE. The risk of developing ACPA and RA for an individual who smokes and carries two copies of the SE is 21-fold higher than for non-smokers who do not carry the SE [80]. Based on these findings, it has been hypothesized that the influence of genes on the susceptibility of RA might be highly dependent on which environmental factors are present [71,79,80].

Other potential environmental risk factors proposed include alcohol intake, coffee intake, vitamin D status, oral contraceptive use and low socioeconomic status, although supporting evidence for these other factors is weak [81].

### 1.4. Epstein-Barr Virus

EBV has been proposed to be involved in the onset of numerous diseases, e.g., mononucleosis and connective tissue diseases such as SLE and RA [82,83,84].

**Figure 2:**
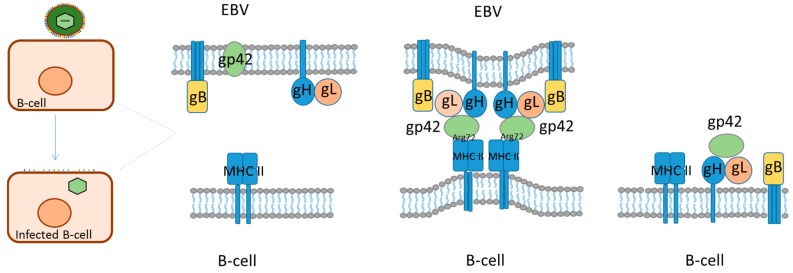
Epstein-Barr B-cell fusion model. Rough sketch of EBV fusing with the cellular lipid bilayer of B-cells. For gp42 to become active, the protein is cleaved N-terminally. Gp42 interacts with gH/gL, and the complex interacts with gB. Gp42 interacts with the β1 domain of MHC-II, which ultimately results in membrane fusion.

EBV is a member of the human herpes virus family. It is an enveloped virus with a 172 kB double-stranded DNA genome coding for 87 proteins and a number of non-coding RNAs. EBV infects pharyngeal epithelial cells upon the first encounter with a host, whereafter it establishes a latent infection in (memory) B-cells [84]. EBV has an elaborate set of glycoproteins (gPs) in its host-derived lipid envelope together with a set of host-derived cellular membrane proteins, which depends on the infected cell. The viral set of gPs constitutes an efficient entry complex and the combination of viral gPs and host-derived envelope proteins enables EBV to switch between B-cells and epithelial cells and to infect several other cell types, including T cells, NK cells and others. EBV furthermore has very efficient immune evasion and exhaustion abilities, including its ability to switch between latent infection, with minimal viral gene expression and lytic infection, with extensive viral gene expression and active virus production. These properties make EBV a constant challenge for the host immune system and it plays an important role in several related diseases, including autoimmune rheumatic diseases. In these diseases, the viral gPs play several roles, notably during entry of target cells, which occurs by an ordered sequence of events. Initially, viral envelope proteins interact with target cell receptors and the viral envelope may then fuse with the plasma membrane (e.g., epithelial cells) or the virus may be endocytosed followed by (pH induced) fusion of the viral envelope with the endosome membrane (e.g., B-cells). In the case of B-cell infection, 5 viral gPs play a major role; gP350/220 interacts with CD21 and gp42 interacts with MHCII, while gB and gH/gL promote membrane fusion (Figure 2). In addition, complement activation products (e.g., C3d) bound to the viral surface may promote interaction by binding to B-cell CR2 (CD21) and the B-cell receptor of memory B-cells may increase interaction, if it has affinity for a viral envelope protein. All this equips EBV with a high tropism for (memory) B-cells and gp42 plays a central role by its interaction with MHCII on B-cells [85,86,87,88,89,90].

#### 1.4.1. Glycoprotein 42, Characteristics and Interactions

EBV gp42 is one of the smallest gPs (223 amino acids) involved in EBV attachment to host B-cells. Although of limited size, this protein is extremely important for B-cell infection, as EBV entry into B-cells requires binding of gp42 to HLA class II. Consequently, virus lacking gp42 can only interact with human B-cells, but cannot infect them [91,92]. Similarly, the amount of gp42 present on the virion determines the cell type that EBV infects [91,92].

EBV gp42 is unique to EBV, but sequence homologs among the closely related primate lymphocryptoviruses and homologs in other herpesviruses exist [93]. The protein contains an N-terminal domain of approximately 100 amino acids and a C-terminal C-type lectin domain (CTLD) [94,95]. While the relatively small, but flexible, N-terminal region interacts with gH/gL, the CTLD interacts with HLA class II. A hydrophobic pocket is located in the CTLD, which appears to be important for its ability to trigger membrane fusion subsequent to HLA class II binding. Mutations in the pocket appear to inhibit fusion, but not binding to gH/gL or HLA, confirming its functional importance in B-cell fusion [96]. Findings by Janz and Haan indicate that the pocket undergoes small structural changes upon interaction with HLA, which could be important for triggering membrane fusion [97,98]. In addition, gp42 contains a transmembrane domain spanning residues 9–29, with its C-terminus on the external side of the membrane [93,99]. 

EBV gp42 occurs in two forms in infected cells, a full-length membrane-bound form and a soluble form, generated by proteolytic cleavage, that is secreted from infected cells due to loss of the N-terminal transmembrane domain. Both the full-length and the secreted gp42 forms bind to gH/g and HLA class II, however, the functional significance of gp42 cleavage is currently unclear [100,101].

Interestingly, gp42 appears to act as a tropism switch that directs fusion with B-cells and inhibits fusion with epithelial cells, a process mediated through its interactions with gH/gL [91]. Similarly, infected B-cells have reduced amounts of gp42 due to sequestration by cellular HLA class II, whereas infected epithelial cells have higher amounts of gp42, as these cells normally do not contain HLA class II [91]. Consequently, virus originating in epithelial cells efficiently infects B-cells, whereas B-cell-derived EBV more efficiently infects epithelial cells [91].

EBV gp42 plays multiple roles during infection, including acting as a co-receptor for viral entry into B-cells by interacting with HLA class II, and binding to EBV gPs gH and gL during the process of membrane fusion, which together with gB constitute the core proteins for EBV entry into cells. gp42 forms a stable, high affinity complex with gH/gL [102]. The residues 36–81 of the N-terminal region of gp42 are critical for the interaction between gp42 and gH/gL. Studies by Kirschner and colleagues have proposed that the N-terminal region interacts with gH/gL by contact through amino acids 44–61 and 67–81 with high molecular affinity in a hairpin-like conformation [103,104]. A current theory is that the gH/gL complex primarily acts as a regulator of gB activation rather than having a direct function in driving membrane fusion [105], which ultimately leads to initiation of membrane fusion.

In contrast to the gH/gL complex, which primarily interacts with the N-terminal domain of gp42, the β-chain of HLA class II binds to the CTLD, more specifically to amino acids 94–221. HLA class II consists of two distinct peptide chains, which non-covalently hetero-dimerize. As a result of this 1:1 interaction, a peptide binding groove is formed by an eight-stranded pleated sheet supporting two helices. However, the interaction between HLA class II and gp42 is not restricted to this binding groove, but to the β-chain of HLA. In fact, gp42 interacts exclusively with the β-1 domain to one side of the peptide binding groove [106]. Studies by McShane and colleagues showed that a soluble form of gp42 generated stable interactions with HLA class II and that especially glutamic acid 46 and arginine 72 in HLA class II were essential for reactivity, which is in accordance to crystal structure analyses of the gp42: HLA-DR1 complex [106,107].

#### 1.4.2. Epstein-Barr Virus as a Contributor to Initiation of Rheumatoid Arthritis

Several studies point to an association between EBV and RA [108,109,110,111,112], thus EBV infection has been considered to be one of the environmental factors that contribute to the onset of RA. It has been demonstrated that individuals with RA display serological signs of EBV infections, e.g., have elevated antibody levels to latent and replicative EBV proteins, e.g., Epstein-Barr viral capsid antigen, early antigen, EBNA-1 and EBNA-2 [109,112,113,114,115]. Moreover, it has been shown that individuals with RA are less efficient in neutralizing autologous EBV-infected cells and prone to have significantly higher numbers of circulating EBV-infected B-cells [108,116,117] and that individuals with RA have elevated viral EBV DNA load compared to controls [109,118,119,120]. Other studies indicate the EBV is associated with RA through molecular mimicry, where antibodies to an EBV-encoded protein (gp110) has sequence homology with the QKRAA motif of the HLA-DR4 [108,121,122]. In addition, individuals with RA have an increased risk of experiencing EBV-associated lymphoma, due to the presence of EBV in a latent stage in the B-cells of RA individuals, supporting the hypothesis that EBV is associated with RA [123,124].

Nevertheless, other studies claim that no association between EBV infection and onset of RA is evident [110,125,126]. For example, findings by Sherina and colleagues, analyzing anti-viral antibodies in relation of ACPAs, smoking HLA-DRB1 alleles and clinical parameters, do not support the hypothesis of EBV involvement in RA onset [126]. These findings are supported by similar studies analyzing antibody levels to several viral proteins [110]. Other findings do not support the hypothesis that EBV infection predisposes to the development of RA, but indicate that EBV infection is associates with other autoimmune diseases such as SLE [125].

These differences between studies describing whether EBV is involved in the onset of RA may be related to differences in cohorts applied and assays used for analysis. Furthermore, the presented studies are conducted using sera from individuals infected with EBV, as up to 99% of humans are infected with EBV, making it very difficult to analyze EBV-negative RA individuals. 

## 2. Discussion

### HLA-DR1 and Gp42 Interaction as a Mediator or EBV Entry and Ultimately Onset of SE-Positive Rheumatoid Arthritis

**Figure 3:**
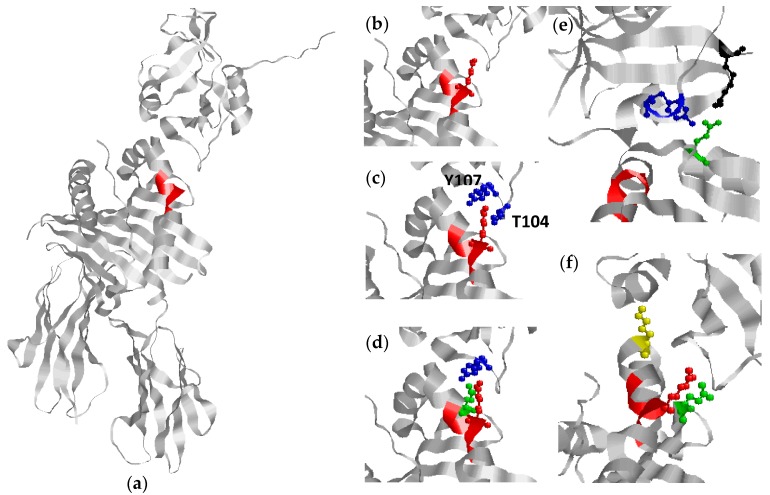
Interactions between gp42 and HLA-DR1: (**a**) crystal structure of the HLA-DR1 and gp42 complex. The shared epitope backbone structure (amino acids 70–74) is colored in red; (**b**) location of Arg72 in the shared epitope; (**c**) interaction between Arg72 (HLA-DR1) and T104 (blue) and Y107 (blue) of gp42; (**d**) interaction between E46 (green) and Arg72 (red) (HLA-DR1) and Y107 (blue) (gp42) through a salt bridge and hydrogen bonding, respectively; (**e**) interaction between E46 (green) (HLA-DRB1) and R220 (black) and Y107 (blue) (gp42); and (**f**) location of E46 (green), I67 (yellow), R72 (red) in HLA-DRB1.

HLA-DR was originally shown to interact with gp42 in an expression library screen for proteins binding to a soluble gp42Fc construct [127]. Subsequent studies demonstrated that the interaction between gp42 and HLA-DR is crucial for EBV infection in B-cells, since monoclonal antibodies to gp42 as well as HLA-DR inhibited B-cell infection in vitro [128]. EBV infects B-cells in vivo through an entry complex, which among others involves the viral gPs, gH, gL, gB and gp42, with gp42 constituting a key factor in activating membrane fusion and hence triggering virus entry (Figure 2) [89,129]. In this process, gp42 interacts with both the viral gH/gL complex and MHC II, which is crucial for EBV entry [127,129,130,131]. Gp42 binds to the β1 domain of the HLA molecule to one side of the peptide binding groove [106]. The specific interaction buries a total surface area of 1002 Å^2^ and constitutes primarily hydrophilic and charged residues. Thorough analysis of the crystal structure of gp42 in complex with HLA-DR1 reveals specific key amino acids (Figure 3), which are characterized as crucial for this interaction. R72 and E46 of HLA-DR1 make extensive interactions with gp42 and substitution analyses confirm that these amino acids are essential for reactivity [107]. E46 is located in the N-terminal end of a strand in the β1 domain at the outer base of the MHC peptide binding groove, whereas R72 is located on the outer face of the second β1 domain α-helix (Figure 3). The crystal structure of the gp42: HLA-DR1 complex reveals that E46 of HLA is directly in contact with R220 and Y107 of gp42 through a salt bridge and a hydrogen bond, respectively, whereas R72 interacts with T104 and Y107 of gp42 through hydrogen bonding [106]. The interaction of R72 with T104 and Y107 forms part of the binding site for E46, which cooperatively link gp42 recognition of E46 and R72, thus a precise positioning of R72 is essential for generating a stable interaction between E46 and R220 of gp42, which has been confirmed by substitution studies [107].

Based on the current description of the EBV gp42-HLA-DRB1 interaction, we hypothesize that R72, which is part of the SE structure located at amino acid positions 70–74 of HLA-DRB1, is directly related to EBV entry. Hence, EBV infection, through specific interactions between gp42 and HLA alleles, might ultimately contribute to the onset of RA. This hypothesis is supported by several findings.

Although the amino acid E46 is not directly related to the SE motif, are the amino acids E46 and R72 of HLA crucial for a stable interaction to gp42 [106,107]. Site-directed mutations of E46 to V, Q or K, reveal that nonfunctional HLA molecules are generated which do not promote EBV entry [130]. Nevertheless, substitution of E46 to aspartic acid does not appear to affect the ability to induce entry, indicating that a negative charge in this position, and hence the presence of a salt bridge, is crucial for interaction in this position. Similarly, R72A and R72E mutants are not able to interact with gp42, which confirm the importance of the extensive interaction of R72 with gp42 in the gp42:HLA-DR1 crystal structure and establish this residue as crucial in mediating interaction and ultimately EBV entry [107]. This may be explained by that in the absence of R72 (or the lack of a precise presentation of R72) no scaffold for E46 is generated, as previously mentioned, and hence the crucial ionic bond between E46 and R220 of gp42 is not established (Figure 3). 

The importance of the E46 and R72 for a stable interaction is confirmed when analyzing HLA alleles, which shows that E46 is completely conserved in HLA-DP sequences and only a single allelic change of E46 is found within DR sequences (to aspartic acid), which has very little effect on EBV entry [130]. Likewise, R72 is predominantly conserved in HLA-DR and completely conserved in HLA-DQ and -DP sequences [106]. These findings are in accordance to that EBV also can use the other two HLA class II isotypes-DP and DQ to gain entry into B-cells [97].

Especially R72, being part of the SE structure, has been found to be essential in predisposing to RA, as illustrated in Table 1. Nevertheless, the residues surrounding R72 are not conserved, but have a profound influence on the MHC II-gp42 interaction by influencing the geometry of R72 and also the stability of the MHC molecule. Studies illustrate that a double mutation of residues 71 and 74 still mediated entry [130]. These findings are in accordance to analyses of the crystal structure of the MHC II: gp42 complex, where no specific interaction between amino acids 70–71 and 73–74 of HLA and gp42 has been identified [106]. Modifying the surrounding amino acids may also affect the peptide structure and ultimately the peptide binding groove. Some studies have suggested that these structural modifications are based more on the charge of the relevant amino acid than on the amino acid sequences and in particular on the charge of the amino acids at positions 70, 71 and 74 [132]. Further studies by Rosloniec and colleagues showed that alleles, which share the RRAA and the KRAA motif, have different binding affinities, although they have the same charge [133]. Thus, physico-chemical properties rather than the specific electric charge appear to be essential for interactions. These findings are in accordance to that the mere presence of R72 not is sufficient for predisposing RA, as HLA-alleles that are negative for the SE motif, but positive for R72, do not predispose to RA. Based on the findings by Ou and Rosloniec, we propose that the crucial amino acids found in the SE motif most likely contribute to ensure a stable α-helix structure, favoring optimal presentation of R72 protruding into the gp42 binding pocket composed by amino acid positions 104–107 of gp42, in combination with providing a peptide scaffold, which is essential for E46 presentation and binding as well (Figure 3). If one or more of these interactions is absent, the HLA allele interacts more weakly with gp42 and supports EBV entry less efficiently [106,107]. This has been proposed by Mullen and colleagues, although it remains to be verified [106]. Moreover, the proposed theory may explain why e.g., the motif DKRAA predisposes to RA, whereas the DRRAA motif does not, as physico-chemical interactions between the amino acids in positions 70 and 71 in the latter are different from the DKRAA motif; although R and K provide the same electric charge, do they contribute differently to the physico-chemical interaction, as the positive charge in R is arranged differently from K due to the specific side chains. Modification in the physico-chemical interactions within the motif may crook the α-helix structure of the SE motif (Figure 1), which may change the protruding presentation of R72 and ultimately reduce the interaction between R72 and gp42. However, structural studies alone may be insufficient to explain completely the role of gp42 and the various RA-promoting and -protecting MHC II forms, since EBV tethering and infection is a highly dynamic process. This view is supported by preliminary molecular dynamics calculations, which indicate that the physical stability of gp42-MHC II complexes cannot alone account for the observed RA susceptibility (unpublished results), although SE residues are clearly crucial for the interaction.

Based on the current findings described in this article, the mentioned studies and observations support the hypothesis that HLA-gp42 interaction in predisposed SE-positive individuals facilitates EBV entry and infection, which ultimately may result in uncontrolled EBV infection (especially in joints, where EBV may drive processes normally restricted to lymph nodes, i.e., antigen uptake and presentation, cytokine release and lymphocyte interactions) and thus in the onset of RA. EBV infects all individuals, as all natural MHC II variants (human) can interact with gp42. However, the interaction with SE-positive MHC II, seems to support EBV entry more efficiently.

The exact mechanism underlying SE-positive RA remains unclear [45,46,47,48,49]. It has been proposed that SE-positive DRB1 alleles confer disease susceptibility through a mechanism that involves alteration of the peripheral T-cell repertoire or through the selective presentation of arthritogenic self or foreign peptides [45,46,47,48,49]. Moreover, it has been proposed that SE-positive HLA alleles exert pathogenic effects through the presentation of citrullinated peptides, which are recognized as non-self by T-cells [50]. Finally, it has been proposed that the SE, analogous to certain domains of class I MHC-molecules [52,53], acts as a ligand that interacts with cell surface calreticulin and activates innate immune signaling [54]. None of the mentioned mechanisms are contradictory in relation to the current hypothesis proposed, and the onset of RA may turn out to involve an interplay between several of these mechanisms.

## Figures and Tables

**Table 1:** Classification of HLA-DRB1 alleles and their role relative to onset of rheumatoid arthritis. Highlighted alleles constitute the most frequently reported alleles associated with rheumatoid arthritis. The risk of developing rheumatoid arthritis is among others associated with the presence of specific amino acids in the amino acid positions 70–74. Crucial is the RAA motif in positions 72–74, but the effect is modulated by the amino acids in positions 71 and 70 as well, where K in position 71 confers the highest risk, R an intermediate risk, and A and E a lower risk. Similarly, the amino acids Q and R in position 70 confer a higher risk than D. Bold alleles represent the most common alleles detected in individuals with rheumatoid arthritis.

Sequence	SE Motif	Alleles	Relative Genotype Risk *	References
QKRAA	+	*04:01, *04:09, *04:13, *04:16, *04:19, *04:21,*14:21	5.9	[32]
DKRAA	-	*13:03	5.9	[32]
QRRAA	+	*01:01, *01:02, *01:05, *04:04, *04:05, *04:08, *04:10, *04:19, *14:02, 14:06, *14:09, *14:13, *14:17, *14:20	3.3	[31,32]
RRRAA	+	*10:01	3.3	[32]
QRRAE	-	*04:03, *04:06, *04:07, *04:11, *04:17, *04:20	1	[33]
RRRAE	-	*09:01, *14:01, *14:04, *14:05, *14:07, *14:08, *14:10, *14:11, *14:14, *14:18	1	[33]
QARAA	-	*13:09, *15:01	1	[33]
QKRGR	-	*03:01, *04:22, *11:07	1	[33]
DRRGQ	-	*07:01	1	[33]
DRRAL	-	*08:01	1	[32]
DRRAA	-	*04:15, *08:05, *11:01, *11:04, *11:05, *11:06, *11:09, *11:10, *11:12, *11:15, *11:18, *11:19, *11:22, *12:01, *13:05, *13:06, *13:07, *13:11, *13:12, *13:14, *13:21, *13:25, *14:22, *16:01, *16:05	1	[31,32]
DERAA	-	*01:03, *04:02, *11:02, *11:03, *11:16, *11:20, *11:21, *13:01, *13:02, *13:04, *13:08, *13:15, *13:17, *13:19, *13:22, *13:23, *14:16, *15:01	1	[31,32]

* Relative to model proposed by Du Montcel et al. [33] when expressing two of the same HLA-DR1 alleles.

## Abbreviations

ACPA	Anti-citrullinated protein antibodies
CD	Cluster of differentiation
CTLA	Cytotoxic T lymphocyte-associated antigen
CTLD	C-type lectin domain
EBV	Epstein-Barr virus
Gp	Glycoprotein
HLA	Human leukocyte antigen
IRF	Interferon-regulating factor
MHC	Major histocompability complex
PTPN22	Protein tyrosine phosphatase, non-receptor type 22
PAD	Peptidyl arginine deiminase
RA	Rheumatoid arthritis
RF	Rheumatoid factor
SE	Shared epitope
SLE	Systemic lupus erythematosus
STAT	Signal transducer and activator of transcription
TRAF	Tumor necrosis factor receptor-associated factor
